# ATLANTIS: a randomised multi-arm phase II biomarker-directed umbrella screening trial of maintenance targeted therapy after chemotherapy in patients with advanced or metastatic urothelial cancer

**DOI:** 10.1186/s13063-020-04283-5

**Published:** 2020-04-19

**Authors:** Ben Fulton, Robert Jones, Thomas Powles, Simon Crabb, James Paul, Alison Birtle, Simon Chowdhury, Syed Hussain, Anna Morris, Eileen Soulis, Paula Morrison

**Affiliations:** 1 Beatson West of Scotland Cancer Centre, 1053 Great Western Road, Glasgow, G12 0YN UK; 2 Institute of Cancer Sciences, Switchback Road, Bearsden, Glasgow, G61 1QH UK; 3 Barts’ Cancer institute, Queen Mary University of London, London, EC1M 6BQ UK; 4 University of Southampton, Faculty of Medicine, 12 University Road, Southampton, SO17 1BJ UK; 5 Cancer Research UK Clinical Trials Unit, Beatson West of Scotland Cancer Centre, 1053 Great Western Road, Glasgow, G12 0YN UK; 6 School of Cancer Sciences, University of Manchester, Oxford Road, Manchester, M13 9PL UK; 7 Guys and St Thomas’ NHS Foundation Trust, Great Maze Pond, London, SE1 9RT UK; 8 Academic Unit of Oncology, University of Sheffield, Beech Hill Road, Sheffield, S10 2RX UK

**Keywords:** Urothelial cancer, Precision medicine, Biomarker

## Abstract

**Background:**

Metastatic urothelial cancer (UC) is the eighth most common cause of cancer death in the UK. Standard first-line treatment, for most patients, is cytotoxic chemotherapy. Although UC is initially sensitive to chemotherapy, relapse is almost inevitable and outcomes are poor; median overall survival is 8 months. Therefore, there is an urgent need for novel therapies to improve outcomes for this patient group.

**Methods:**

ATLANTIS is a randomised phase II umbrella-design screening trial of maintenance therapy in biomarker-defined subgroups of patients with advanced UC. The primary end point is progression-free survival, and the study involves over 30 UK cancer centres.

**Discussion:**

ATLANTIS is the first study in the UK to employ a precision-medicine approach to patients with UC for maintenance treatment. Agents with a positive efficacy signal will proceed to randomised phase III trials to confirm the activity of novel, biologically stratified therapies in UC.

**Registration:**

ATLANTIS trial EudraCT number 2015–003249-25. ISRCTN25859465.

## Background

Urothelial cancer (UC) is the eighth most common cause of cancer-related death in the UK. Around 5300 patients died from UC in the UK in 2016 (Cancer Research UK, 2017). Cytotoxic platinum-based doublet chemotherapy is routinely used for metastatic or locally advanced disease in the first-line setting [1, 2]. Although the majority of patients derive benefit, relapse is almost inevitable and occurs an average of 4 months after completion. Once relapse has occurred, survival and quality of life are often poor; median progression-free survival (PFS) and overall survival (OS) are short: 2 and 8 months respectively [3]. In recent years, immune checkpoint inhibitors, which can benefit around 20% of patients with durable responses and proven survival advantage, have found a role in second-line treatment after failure of platinum-based chemotherapy [4, 5]. Their role in first-line treatment is currently limited to patients who have high programmed death-ligand 1 (*PD-L1*) expression and who are not suitable for platinum-based chemotherapy [6, 7]. However, there are still a majority of patients with advanced UC who do not derive significant benefit from immune checkpoint inhibitors and whose subsequent treatment options are very limited. Second-line chemotherapy may be used, but response rates are low and benefit compared with best supportive care is uncertain, although recent data suggest that the combination of docetaxel and the vascular endothelial growth factor (*VEGF*) targeted antibody ramucirumab may provide modest benefits in selected patients in the second-line setting [8].

Therefore, there is a clear need for new effective treatments for patients with advanced UC. The molecular heterogeneity of UC suggests that patients may be well served by a precision-medicine approach. Testing new drugs in combination with first-line chemotherapy is often challenging because of toxicity of combinations in this patient group [9]. Studies in the second-line setting have historically been limited by the high symptom burden and poor prognosis for patients. Therefore, maintaining clinical benefit after first-line chemotherapy may be an attractive way to improve outcomes for patients with advanced UC.

Maintenance therapy after first-line chemotherapy is an opportunity for novel drug development in advanced UC. This was demonstrated in the UK NCRI LAMB trial, which completed accrual in 2013 after having screened 520 patients and randomly assigned 221 patients with epidermal growth factor–expressing urothelial tumours from over 40 UK sites between lapatinib and placebo (ClinicalTrials.gov Identifier: NCT00949455). In that trial, lapatinib did not prolong survival or PFS in any of the defined subgroups, confirming observation alone as the standard of care in this patient group [10]. The study did reinforce the proof of concept that such a trial would be acceptable to patients and could be successfully delivered in the UK.

## Methods and study design

**Fig. 1:**
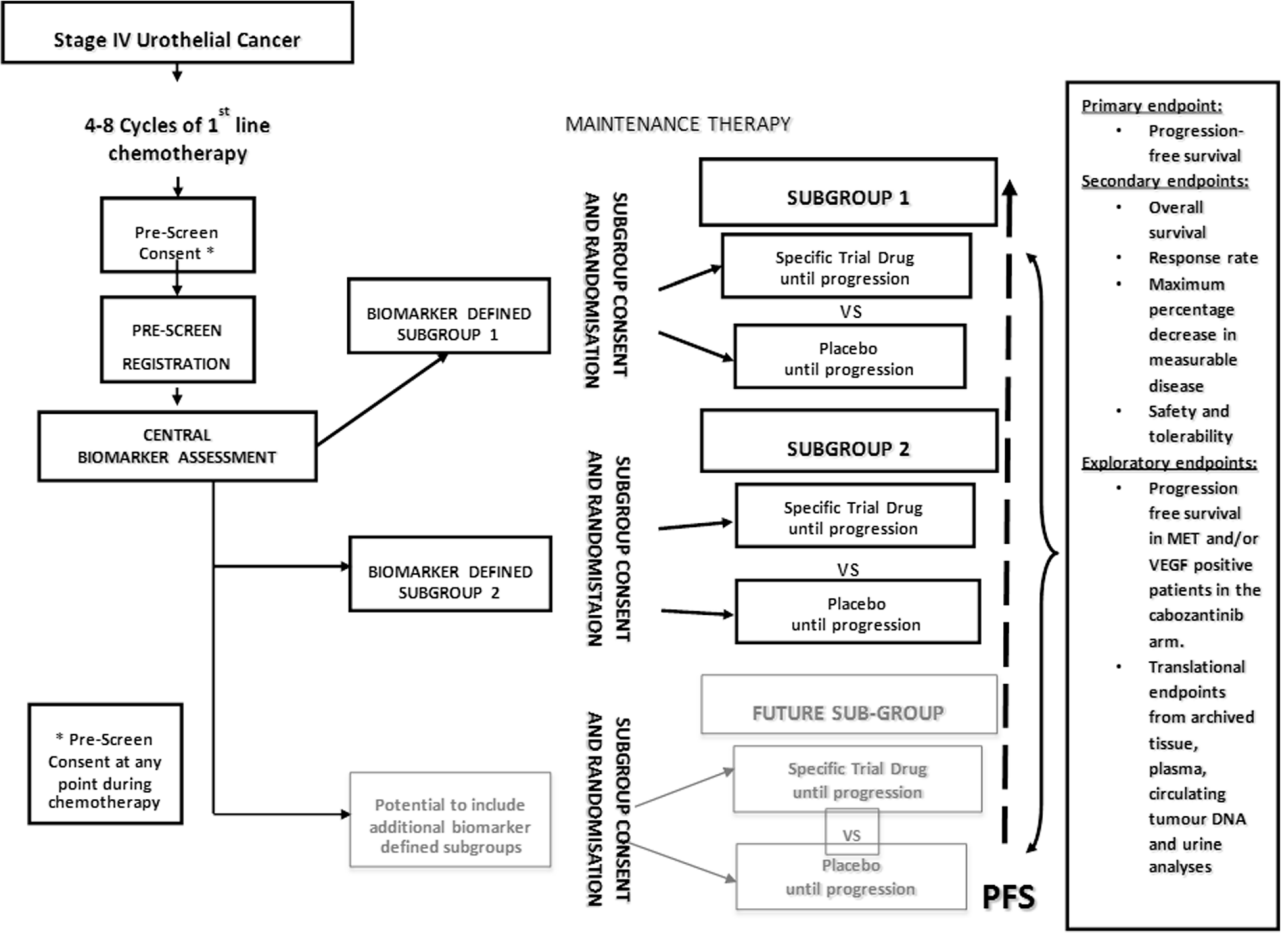
Study design for patients in ATLANTIS trial. *Abbreviations*: *MET* MET proto-oncogene, *PFS* progression-free survival, *VEGF* vascular endothelial growth factor

ATLANTIS is a multi-centre randomised phase II signal-searching umbrella-design screening trial of targeted novel agents in biomarker-defined subgroups (Fig. 1). Multiple novel agents will be used in parallel and patients will be entered into ATLANTIS subgroup studies dependent on tumour biomarker profile. The control arm for each comparison will be matched placebo, and comparison will be double-blind where possible.

### Trial population

The target population in ATLANTIS consists of patients with metastatic or locally advanced UC (T4b and/or N1–3 and/or M1) who are not being considered for radical therapy. Patients must have achieved an objective response or stable disease, according to local radiology review, after 4–8 cycles of first-line chemotherapy. Patients must be able to start maintenance treatment within the study at least 3 but no more than 10 weeks after completion of their first line of chemotherapy for metastatic or locally advanced disease. Biomarker analysis of archival tissue to determine ATLANTIS biomarker-defined subgroups can occur any time after the diagnosis of UC, after appropriate consent has been given.

### Study objectives and end points

The principal research question is whether molecularly targeted maintenance therapy after chemotherapy can delay the time to progression in molecularly selected patients with advanced UC. ATLANTIS will thereby establish initial evidence of activity for the novel drug/biomarker combinations used in order to justify further phase III validation. A number of drugs will be tested, each compared with placebo or (where this is not feasible) observation alone. Treatment will be allocated on the basis of molecularly defined subgroups of patients (where laboratory/clinical evidence to support such enrichment is clear) or in a manner that allows exploration of, or provides initial evidence for, predictive biomarkers. Of note, it is anticipated that in most cases the biomarker for a particular arm of the trial will itself be experimental and not yet prospectively validated.

The primary end point is PFS. This has been chosen as it is largely objective and the majority of patients with UC display progression in accordance with RECIST 1.1 criteria. PFS is also clinically meaningful as the progression after first-line chemotherapy represents the transition to the lethal stage of the disease and often the requirement for further systemic therapy.

**Fig. 2:**
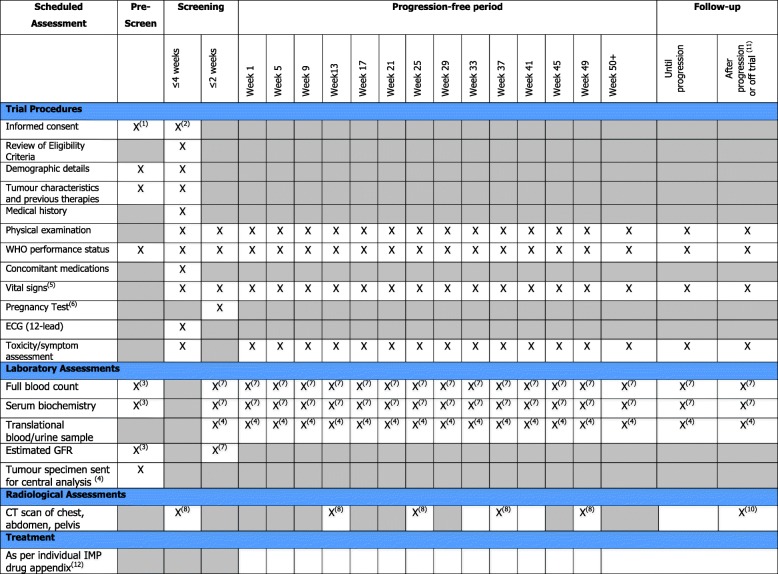
Standard Protocol Items: Recommendations for Interventional Trials (SPIRIT) figure: Schedule of Assessments for ATLANTIS trial. (1) Patients should have signed and dated informed consent forms for pre-screening. (2) Each patient must have signed and dated both informed consent forms for pre-screening biomarker testing and full trial screening before engaging in any trial-related procedures. All screening evaluations must be completed before the patient is randomly assigned to receive trial drug or placebo. (3) Patient characteristics will be collected at pre-screening. These data should be collected only if they are available from data collection during the previous 6 weeks as part of standard care. No additional blood tests should be performed during pre-screening purely for the trial. (4) Tumour samples and all other translational samples should be sent to the attention of Charlotte Ackerman for centralised pre-screening or confirmation. If there is any tissue left from biomarker testing, it will be stored in the Orchid Tissue Bank for future translational research. Individual samples will be returned at the end of the trial on request. Samples will be processed in accordance with the ATLANTIS lab manual. (5) Weight, height, pulse and blood pressure. (6) Human chorionic gonadotropin (HCG) results must be obtained and reviewed before the first dose of Investigational Medicinal Product (IMP) is administered for women of child-bearing potential. (7) Haematology, including full blood count with white blood cell count, absolute neutrophil count, platelet count and haemoglobin. Biochemistry, including sodium, potassium, aspartate aminotransferase/alanine aminotransferase (AST/ALT), alkaline phosphatise, lactate dehydrogenase, bilirubin, creatinine, protein and albumin. (8) All patients should have abdominal and pelvic computed tomography (CT) or magnetic resonance imaging (MRI) plus either chest x-ray (postero-anterior and lateral views) or chest CT scan. If known or thoracic metastases are seen on chest x-ray, patients must have a thoracic CT scan. Patients should have baseline scanning every 12 weeks until week 49; following this, scans should be carried out at the discretion of the individual clinician. (9) Patients who come off the trial should have tumour assessments within 4 weeks of coming off trial drug/placebo, irrespective of whether the patient is still being followed up for progression. (10) Patients who come off the trial should have tumour measurements where they have not been completed within the past 4 weeks. This includes abdominal and pelvic CT or MRI plus either chest x-ray (postero-anterior and lateral views) or chest CT scan. If known or thoracic metastases are seen on chest x-ray, patients must have a thoracic CT scan. Patients who stop treatment for whatever reason before progressive disease is documented will continue to have scans at 12-weekly intervals as previously. (11) Follow-up visits after progression will continue at the investigators’ discretion until death. Future treatment and cause of death must be recorded on the case report form. (12) Frequency of treatment visits will vary within the different treatment arms; please see individual IMP drug appendices for this information. *Abbreviations*: *ECG* electrocardiogram, *GFR* glomerular filtration rate, *WHO* World Health Organization

The secondary end points in ATLANTIS are OS, response rate, maximum percentage decrease in measurable disease, safety and tolerability. Exploratory end points are PFS in biomarker-defined subgroups other than those used for selection. Exploratory research hypotheses are embedded in the primary purpose of the trial. As such, all patients must provide adequate tissue for biomarker analysis prior to participation in the trial. This tissue collection will provide a bio-resource for future research relating to UC. The trial also provides a generic umbrella-design framework allowing new drugs to be introduced by amendment in the future. The SPIRIT (Standard Protocol Items: Recommendations for Interventional Trials) figure for ATLANTIS trial is shown in Fig. 2.

### Current biomarker-defined arms in ATLANTIS

The design of ATLANTIS allows for the addition of further biomarker-defined subgroups throughout the lifetime of the trial. At initiation, ATLANTIS was exploring a single drug (cabozantinib) but other comparisons, each with associated Investigational Medicinal Product (IMP), have been added since the trial began:

#### Cabozantinib

A wide body of pre-clinical evidence supports the relevance of hepatocyte growth factor, the ligand for *MET*, and *VEGF* as anti-cancer targets in patients with UC [11]. Cell-line data show that *MET* is associated with tumour proliferation and growth [12]. *VEGF* has also been demonstrated to be over-expressed in UC cell lines and associated with tumour proliferation [13]. Therefore, the combination of *VEGF* and *MET* inhibition is an attractive field in patients with UC. Cabozantinib is a multi-kinase inhibitor including *MET* and *VEGF*. It has been demonstrated to have significant activity and is licensed in Europe to treat medullary thyroid and renal cancers [14]. It has shown early signs of activity in an ongoing phase II trial in patients with advanced UC [15]. There is currently a lack of data on the expression of *MET* or *VEGF* as a biomarker for cabozantinib activity. For this reason, all patients in ATLANTIS and other ongoing studies have not selected patients on the basis of *MET* expression. Therefore, patients in this study will potentially be eligible for the cabozantinib/placebo arm if their tumour does not over-express any current ATLANTIS target biomarker or they are not deemed suitable to enter a biomarker-defined arm of the study.

#### Rucaparib

There is emerging evidence of a subgroup of patients with UC exhibiting a DNA repair deficiency phenotype resulting in defects in a variety of genes, including* BRCA1/2*, *BAP1*, *PALB2*, *FANCD2* and *ERCC2* [16, 17]. These DNA repair gene defects predict for benefit following cisplatin-based chemotherapy in UC, implying that a switch to maintenance therapy strategy for poly (ADP-ribose) polymerase (*PARP*) inhibition after prior chemotherapy may allow for enrichment of a ‘BRCA-like’ subgroup. *PARP* inhibition has demonstrated activity against multiple UC cell lines and xenografts. Evidence supports the development of *PARP* inhibitors in patients with either germline or somatic BRCA mutations and, in addition, a wider selected group with evidence of homologous recombination deficiency (*HRD*)-associated tumours [18]. *PARP* inhibitors have demonstrated compelling evidence of efficacy in patients with relapsed high-grade serous ovarian cancer demonstrating germline or somatic *BRCA* mutation [19]. The concept of synthetic lethality was confirmed in proof-of-concept studies in patients with *BRCA*-associated tumours where data suggest that *BRCA* mutation is significant but not required.

These data imply that *BRCA* mutation or HRD-associated UC patient subgroups (or both) would be suitable for investigation of a *PARP* inhibitor. Rucaparib is an orally bioavailable small-molecule inhibitor of *PARP1*, *PARP2* and *PARP3*. Non-clinical evaluation of rucaparib has demonstrated potent inhibition of *PARP* enzymes and sensitivity to *BRCA1/2* homozygous mutant cell lines. Patients in ATLANTIS whose tumour is positive for a composite ‘HRD biomarker’ of alterations to a list of relevant DNA repair genes or high percentage genomic loss of heterozygosity (LOH) at pre-screening (or both) will potentially be eligible to receive either rucaparib or placebo in a double-blind fashion. LOH is a form of genomic alteration associated with HRD and is characterised by the loss of one copy of a gene or chromosomal region.

#### Enzalutamide

Previous *in vivo* and *in vitro* data have demonstrated that the androgen receptor (AR) is a potential anti-cancer target receptor in patients with UC [20, 21]. Unpublished data from tissue microarray in patients tested within the LAMB study demonstrated that 30% of UC tumours over-express the AR. This was also associated with poorer prognosis, raising potential clinical benefit of targeted agents against AR. Enzalutamide is a potent AR antagonist which, unlike earlier generations of anti-androgen therapies, has no known agonistic effect on AR and is known to be active in men with prostate cancer who have previously failed conventional androgen deprivation therapy. The drug is well tolerated even with prolonged administration [22]. Patients in ATLANTIS whose tumour is found to over-express the AR on immunohistochemical analysis will potentially be eligible to receive either enzalutamide or placebo in a double-blind fashion.

### Trial analysis plan

The design of each ATLANTIS subgroup is based on a randomised phase II screening design to detect a certain level of improvement in PFS with the novel drug compared with matched placebo with 90% power at the 20% one-sided level of statistical significance or equivalently with 80% power at the 10% level of statistical significance. At the end of each subgroup analysis, if the observed PFS difference in favour of the novel agent is statistically significant, this may be a signal that a subsequent phase III trial is warranted. Within each subgroup of ATLANTIS, randomisation (1:1) will be stratified via minimisation factors according to the following: cisplatin versus non-cisplatin primary chemotherapy, Eastern Cooperative Oncology Group (ECOG) score, best response to first-line chemotherapy, progression during the final three months of chemotherapy, presence of visceral metastases, presence of measurable disease at trial entry and investigational site.

The data analysis will be on an intention-to-treat basis within each trial subgroup. PFS will be compared between the novel agent and placebo in each study subgroup within the context of a Cox model incorporating the baseline minimisation factors, based on a randomised phase II screening design to detect improvement in PFS with the novel drug compared with placebo/observation. The *P* value for the observed hazard ratio will be determined from the Cox model in each subgroup. If the observed PFS difference in favour of the novel agent is statistically significant at the 10% one-sided level, this will be deemed a clear signal that a subsequent phase III trial is warranted. A result significant at the 20% one-sided level would require further evidence in terms of improvement, such as reduction in size of measurable disease. A Mann–Whitney *U* test will be used for this comparison. This decision-making process follows a three-outcome-type design. The OS and PFS will be illustrated by using Kaplan–Meier plots. Response rates will be compared within subgroups in the context of a logistic model incorporating minimisation factors. The worst toxicity grades experienced during chemotherapy will be compared by using the Mann–Whitney *U* test.

The study data will be reviewed roughly annually by an independent data monitoring committee (IDMC). In each ATLANTIS subgroup, there is a non-binding test for futility after half the PFS events have occurred based on a Lan–DeMets monitoring boundary with an O’Brien–Fleming stopping rule. The IDMC will also review toxicity, treatment delivery and compliance data. Recommendations will take into account all available data as well as formal futility comparison for PFS within each trial subgroup.

### Sample size

Patients within the cabozantinib subgroup will be randomly assigned to cabozantinib/placebo in a 1:1 ratio. The median PFS on the placebo arm is estimated to be 6 months. The comparison between arms is designed to detect an improvement in median PFS of 9 months with cabozantinib, which corresponds to a hazard ratio of 0.67. This is appropriate for a new agent within an untargeted population and requires 114 PFS events, so recruitment to this subgroup will incorporate 140 patients over about 24 months. This target provides more than 90% power (10% one-sided) to detect between the study arms a difference in reduction of measurable disease corresponding to a standardised effect size of 0.5. Patients allocated to the rucaparib subgroup will be randomly assigned to rucaparib/placebo in a 1:1 ratio. A hazard ratio for this subgroup of 0.5 is targeted on the basis of the striking effect seen with PARP inhibitors in a similar setting with ovarian cancer using HRD biomarker for patient selection. This requires 39 PFS events, which can be obtained by recruiting 48 patients. This recruitment number also provides more than 80% power (10% one-sided) to detect a difference of reduction in size of measurable disease corresponding to standardised effect size of 0.7.

Patients allocated to the enzalutamide subgroup will be randomly assigned to enzalutamide/placebo in a 1:1 ratio. The median PFS on the placebo arm is estimated to be 4 months. The comparison between arms is designed to detect an improvement in median PFS of 6.7 months with enzalutamide, corresponding to a hazard ratio of 0.6. This size of difference is appropriate in a targeted agent in the target population. This requires 72 PFS events, so recruitment to this subgroup will incorporate 80 patients. This number also provides 90% power (10% one-sided) to detect between the arms a difference in the reduction in size of measurable disease corresponding to standardised effect size of 0.65.

## Discussion

Recent studies of second-line therapy, particularly immune checkpoint inhibitors, in patients with UC have demonstrated a clinical benefit for a small proportion of patients. The emerging treatment landscape of UC is also increasingly incorporating these therapies in first-line clinical trials. However, there remains a need to improve outcomes for patients with this challenging disease. ATLANTIS offers a novel approach to previous studies in this arena by offering biomarker-defined selection of maintenance therapy after chemotherapy in patients with UC. The primary research question of this signal-searching multi-arm phase II study is whether targeted maintenance therapy after chemotherapy can delay the time to progression in molecularly selected patients with UC. This would establish clinically relevant evidence of activity in molecularly defined subgroups for the novel drug used. The study design will investigate cabozantinib, enzalutamide and rucaparib in this setting but also provide a generic framework that will allow new treatments to be introduced into the study in the future with prospective stratification based on a molecular target.

At present, there are no targeted therapies with proven activity in UC. Pre-clinical data suggest that there are a number of eligible targets, but the number of randomised trials performed to test these hypotheses has been small. The reasons behind this remain unclear, but low levels of clinical activity in unselected patients with UC in non-randomised phase II trials do not support progression to phase III trials. Another explanation is that the combination of targeted therapy and standard chemotherapy is poorly tolerated in this patient population. ATLANTIS will be a leading global study in the development of personalised targeted therapy for patients with UC.

Such a positive randomised phase II study would be a significant breakthrough in UC and may lead to randomised phase III trials in both the metastatic and adjuvant setting. If a cohort of ATLANTIS is positive, it would be anticipated to lead to a randomised phase III study of maintenance therapy in the appropriately selected UC population. There is also an unmet need for adjuvant therapy in patients with high-risk muscle invasive bladder cancer, and a positive signal from ATLANTIS would also support a randomised phase III trial in this patient group with appropriate molecular selection.

Patients in ATLANTIS are asked to consent to the collection of surplus tissue for translational research. Translational research hypotheses are embedded in the primary purpose of this trial, the aim of which is to demonstrate efficacy of predictive and appropriate drug interventions in UC. As such, all patients must provide adequate tissue for biomarker expression prior to trial participation. This will also provide a bio-resource for future research in UC.

The ATLANTIS trial reflects an exciting innovation in front-line precision cancer medicine for patients with advanced UC. The study design, with a biomarker-negative arm, allows all patients who enter pre-screening to potentially be able to take part. The adaptive design also allows maximal opportunity to detect an efficacy signal and rapid inclusion of new hypotheses. The engrained translational research components have the potential to resolve some of the unanswered questions and push new frontiers in the management of this challenging disease.

## Trial status

At the time of publication, ATLANTIS was open to recruitment across 32 UK cancer centres. Trial recruitment began in November 2016, and the estimated first subgroup recruitment end date is December 2020. The current ATLANTIS protocol is version 2.4 (21 June 2019).

## Supplementary information

**Additional file 1.** Standard Protocol Items: Recommendations for Interventional Trials (SPIRIT) 2013 Checklist: Recommended items to address in a clinical trial protocol and related documents.

## Data Availability

Not applicable.

## Abbreviations

AR: Androgen receptor
BAP1: BRCA-1 associated protein-1
BRCA: Breast cancer susceptibility protein
ERCC2: Excision repair cross-complementation group-2
FANCD2: Fanconi anaemia group D2 protein
HRD: Homologous recombination deficiency
IDMC: Independent data monitoring committee
LOH: Loss of heterozygosity
MET: MET proto-oncogene
OS: Overall survival
PALB2: Partner and localizer of BRCA2
PARP: Poly (ADP-ribose) polymerase
PFS: Progression-free survival
UC: Urothelial cancer
VEGF: Vascular endothelial growth factor
